# Non-pharmacological Approaches to Depressed Elderly With No or Mild Cognitive Impairment in Long-Term Care Facilities. A Systematic Review of the Literature

**DOI:** 10.3389/fpubh.2021.685860

**Published:** 2021-07-16

**Authors:** Carla Gramaglia, Eleonora Gattoni, Debora Marangon, Diego Concina, Elena Grossini, Carmela Rinaldi, Massimiliano Panella, Patrizia Zeppegno

**Affiliations:** ^1^Department of Translational Medicine (DIMET), Università del Piemonte Orientale, Novara, Italy; ^2^Psychiatry Ward, Maggiore della Carità University Hospital, Novara, Italy

**Keywords:** aging, elderly, depression, treatment, long term care facility, nursing home, systematic review

## Abstract

**Introduction:** Compared to old people who live at home, depressive symptoms are more prevalent in those who live in long-term care facilities (LTCFs). Different kinds of non-pharmacological treatment approaches in LTCFs have been studied, including behavioral and cognitive-behavioral therapy, cognitive bibliotherapy, problem-solving therapy, brief psychodynamic therapy and life review/reminiscence. The aim of the current review was to systematically review non-pharmacological treatments used to treat depressed older adults with no or mild cognitive impairment (as described by a Mini Mental State Examination score > 20) living in LTCFs.

**Methods:** A research was performed on PubMed and Scopus databases. Following the Preferred Reporting Items for Systematic Reviews and MetaAnalyses (PRISMA) flowchart, studies selection was made. The quality of each Randomized Controlled Trial was scored using the Jadad scale, Quasi-Experimental Design studies and Non-Experimental studies were scored based on the Newcastle-Ottawa Scale (NOS)

**Results:** The review included 56 full text articles; according to the type of intervention, studies were grouped in the following areas: horticulture/gardening (*n* = 3), pet therapy (*n* = 4), physical exercise (*n* = 9), psychoeducation/rehabilitation (*n* = 15), psychotherapy (*n* = 3), reminiscence and story sharing (*n* = 14), miscellaneous (*n* = 8).

**Discussion and Conclusion:** Despite mixed or negative findings in some cases, most studies included in this systematic review reported that the non-pharmacological interventions assessed were effective in the management of depressed elderly in the LTCFs context. Regrettably, the limitations and heterogeneity of the studies described above hinder the possibility to generalize and replicate results.

## Introduction

According to the World Health Organization (WHO), between 2015 and 2050 the proportion of the older adults (>60 years old) is estimated to almost double to 22%. Among the general older population, depression occurs in 7% and it accounts for 5.7% of Years Lived with Disability (YLDs). Under the label of “depression,” different diagnoses can be found in older adults: major depressive disorder (MDD), bipolar disorder (BD), minor depression, mood disorders related to a general medical condition, bereavement, adjustment disorder, substance-induced mood disorder, and dementia with depressed mood. Overall, in this selected population depression is often underdiagnosed and undertreated, sometimes even neglected. Moreover, older depressed people have poorer functioning compared to those with other chronic medical conditions (e.g., lung disease, hypertension or diabetes) (1).

A particularly frail portion of older adults is represented by those living in nursing homes and assisted living facilities. Compared to old people who live at home, depressive symptoms are more prevalent in those who live in long-term care facilities (LTCFs) (2). Teresi et al. (3) reported a prevalence of major depressive disorder and minor depression of 14.4 and 16.8%, respectively, in nursing home residents. It is also noticeable that depression raises risk for medical morbidity and poor health outcomes in older adults and it can be a predictor for long-term risk of increased disability, heart disease, dementia, mortality, and suicide (4, 5).

Briefly, depressive disorders in older adults represent a heterogenous and complex phenomenon and a diagnostic challenge, due to not recognized or underestimated symptomatology, correlation with cognitive impairment or other severe medical conditions, overlapping effects of pharmacological treatments—both psychopharmacological and other—and finally peculiar existential or contextual problems, with particular regard to the nursing homes setting. This complexity can be found also in treatment approaches, especially non-pharmacological ones in nursing homes (6, 7).

Different kinds of non-pharmacological treatment approaches in LTCFs have been applied, with results suggesting a high variability on the subpopulations of elderly involved, settings, treatment models and outcomes (8–10). Several psychotherapeutic approaches have been studied and described in previous reviews, including behavioral and cognitive-behavioral therapy, cognitive bibliotherapy, problem-solving therapy, brief psychodynamic therapy, and life review/reminiscence (11–14), Reminiscence Therapy (RT) (15), light therapy (16), music-art-creative therapy (17), and pet therapy (18). Nonetheless, only few of these reviews specifically focused on the specific population of non-cognitively impaired elderly living in nursing homes.

The aim of the current review was to systematically review non-pharmacological treatments used to treat depressed older adults with no or mild cognitive impairment (as described by a Mini Mental State Examination [MMSE] score > 20) living in LTCFs, in order to offer an up-to-date synthesis of the available literature about this topic.

## Methods

Our research question was the following: Are non-pharmacological approaches effective in addressing depression in elderly people with no or mild cognitive impairment residing in LTCFs?

Our PICO (20) was:

**Table:** 

**P**opulation	Elderly people (age over 65 years old) residing in LTCFs, with no or mild cognitive impairment
**I**ntervention	Any non-pharmacological approach
**C**omparison	No-intervention group (when applicable)
**O**utcome	Depression, as assessed by validated psychometric measures

PubMed and Scopus databases were searched on 03/05/2020 with the following search strings:

PubMed database:

(((((“aged”[MeSH] OR “aging”[MeSH] OR “Aged, 80 and over”[Mesh] OR “old age people” OR elder^*^ OR adult^*^ OR retired OR ancient^*^))))) AND (((((((((((((((“Assisted Living Facilities”[Mesh]) OR “Community Health Nursing”[Mesh]) OR “Group Homes”[Mesh]) OR “Halfway Houses”[Mesh]) OR “Health Facility Environment”[Mesh]) OR “Homes for the Aged”[Mesh]) OR “Institutionalization”[Mesh]) OR “Long-Term Care”[Mesh]) OR “Nursing Care”[Mesh]) OR “Nursing Homes”[Mesh]) OR “Rehabilitation Centers”[Mesh]) OR “Rehabilitation Nursing”[Mesh]) OR “Residential Facilities”[Mesh]) OR “Housing for the Elderly”[Mesh]) OR “Geriatric Nursing”[Mesh])) AND ((depression [MeSH] OR depression [text word] OR depress^*^ [text word] OR depressive symptoms [text word] OR emotional depression [text word] OR depressive disorder [MeSH] OR depressive disorder [text word] OR depressive disorder, major [MeSH] OR major depression [text word] OR MDD [text word] OR major depressive disorder [text word]))

Scopus database:

(ALL (“Assisted Living Facilities” OR “Community Health Nursing” OR “Group Homes” OR “Halfway Houses” OR “Health Facility Environment” OR “Home(s)^*^ for the Aged” OR “Institutionalization” OR “Long-Term Care” OR “Nursing Care” OR “Nursing Homes”)) AND (ALL (“aged” OR “aging” OR “Aged, 80 and over” OR “old age people” OR elder^*^ OR adult^*^ OR retired OR ancient^*^)) AND (TITLE-ABS-KEY (“Depression”) OR TITLE-ABS-KEY (“Depressive symptoms”) OR TITLE-ABS-KEY (“Emotional depression”) OR TITLE-ABS-KEY (“Depressive disorder”) OR TITLE-ABS-KEY (“Major depression”) OR TITLE-ABS-KEY (“MDD”) OR TITLE-ABS-KEY (“Major depressive disorder”) OR TITLE-ABS-KEY (“Depress^*^”))

**Figure 1:**
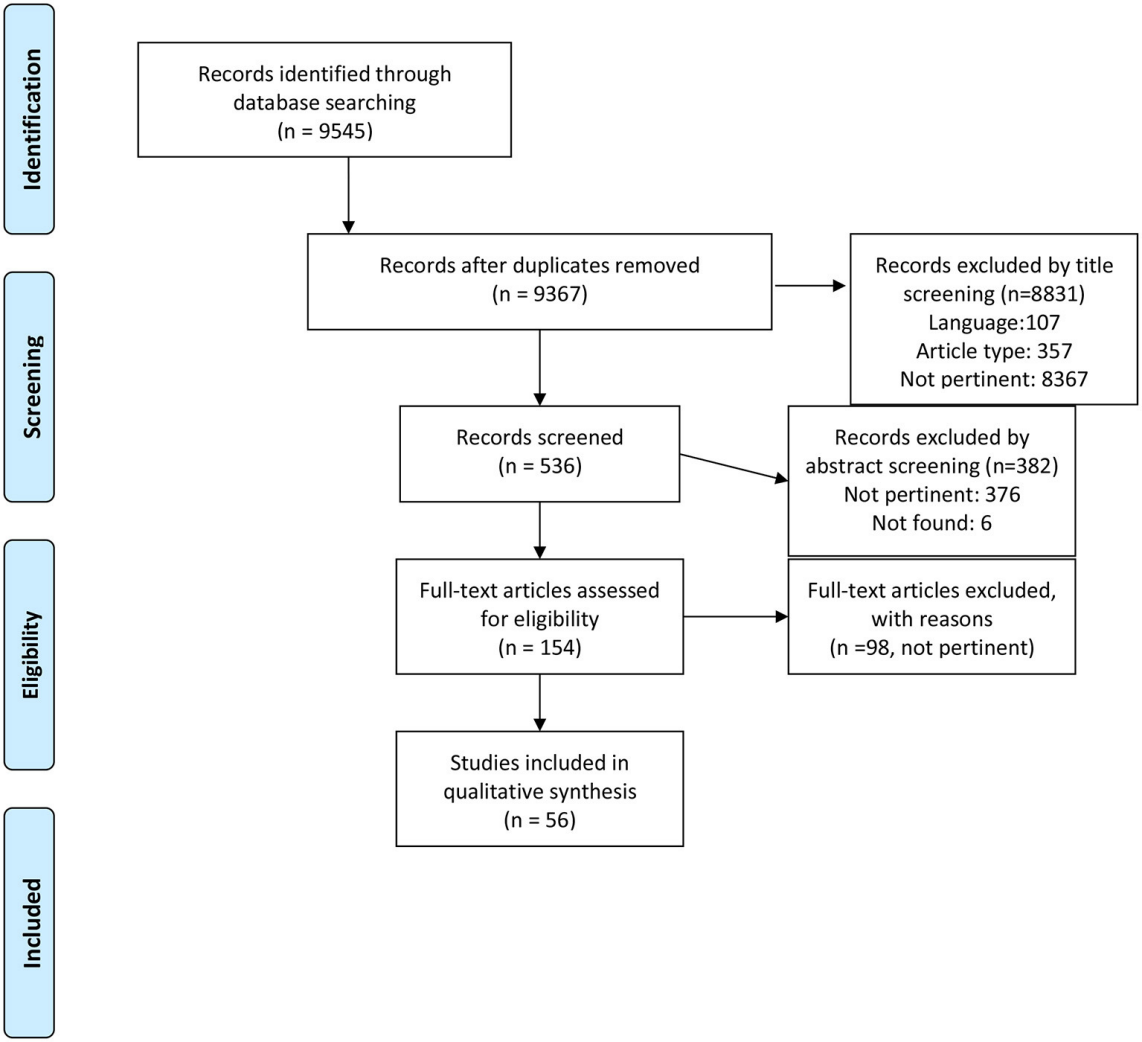
PRISMA 2009 flow diagram. From: Moher et al. (19).

Following the Preferred Reporting Items for Systematic Reviews and MetaAnalyses (PRISMA, 2009) flowchart (19) (Figure 1), studies selection was made from 04/05/2020 to 10/07/2020, screening titles first, then abstracts and eventually the full texts of the articles. Two independent reviewers (D.M., E.G.) assessed the articles identified by the search strings (see above); a third reviewer (C.G.) resolved any discrepancies that emerged between the reviewers.

The following inclusion/exclusion criteria were established for the selection of studies:

Patients' age over 65 years old; patients living in nursing home residence or similar structures (LTCFs); diagnosis of depression, either based on clinical assessment or on the administration of questionnaires or scales; no or mild cognitive impairment (as described by a MMSE score > 20, or by a score on the Mini-Cog ≥ 3, or similar results among other tests for the assessment of cognitive impairment).

Literature reviews, meta-analyses, case reports, editorials, book comments and chapters, articles not in English language were excluded from this review work.

For each study, the following information was tabulated: author and year of publication, sample size, demographic data (mean age, gender), dropout rate, type of study design (RCT: Randomized Controlled Trial; QED: Quasi Experimental Design; NE: Non-experimental), residential care type, measures for dementia, measures for depression, intervention performed (type of intervention, qualification of the intervention provider, setting, monotherapy/augmentation), main findings. Finally, the quality of each RCT was scored using the Jadad modified scale,^1^ QED and NE were scored on the basis of the Newcastle-Ottawa Scale (NOS) (see text footnote 1). The Jadad modified (maximum 8 points) is a scale which assess the quality of studies (randomization, double-blinding, withdrawals and dropouts, inclusion and exclusion criteria, statistical analyses used) (see text footnote 1). The NOS (maximum 9 points) is a scale used for assessing the quality of QES and NE evaluating selection and comparability of cohorts and assessment of outcome (24).

In order to estimate the effect size of each single intervention, we calculated standardized mean differences (SMDs) with 95% CIs to assess depression severity across different depression scales. We used Hedges' adjusted g statistic to calculate SMDs (it is very similar to Cohen's d statistic, but includes an adjustment for small sample bias). Standard mean differences were calculated using RevMan 5.4.

## Results

### Study Selection and Characteristics

According to the inclusion/exclusion criteria described above, the review eventually included 56 full text articles; according to the type of intervention, studies were grouped in the following areas: horticulture/gardening (*n* = 3), pet therapy (*n* = 4), physical exercise (*n* = 9), psychoeducation/rehabilitation (*n* = 15), psychotherapy (*n* = 3), reminiscence and story sharing (*n* = 14), miscellaneous (*n* = 8, for those studies which did not seem to match any of the previous categories) (See the Synthesized findings section below for more details).

Sample size varied from 5 to 403 participants (we decided not to include case studies with <5 participants) and eligibility criteria were highly heterogeneous as well. Regarding methodological approach, 93% (*n* = 52) of the studies examined by this review were either Randomized Controlled Trials (RCT, *n* = 30), or had a Quasi-Experimental Design (QED, *n* = 22); 4 studies were had a Non-Experimental design (NE).

Regarding validated psychometric measures, the GDS was the most used one in the studies included in the current review in its 30-item version (25–37), but even more in its short (15-item) version (22, 23, 38–59).

Most of the studies considered in our systematic review were performed in the Asian continent (41%); with more detail, 14 articles were from Taiwan (21, 30, 32, 36, 49, 50, 52, 57, 58, 60–64). The other studies were distributed as follows: *n* = 13 in Europe (33, 38–40, 43, 47, 54, 59, 65–69), *n* = 17 in America (25, 27–29, 31, 34, 35, 41, 44, 48, 70–76), *n* = 3 in Oceania (26, 42, 77); no study was performed in Africa. All the articles dealing with horticulture were conducted in the Asian continent, while 75% of the articles dealing with pet therapy came from Italy (38–40).

Regarding treatment duration, although data were missing in some studies, interventions lasted mostly for 4–8 weeks. With more detail, 13 studies (21, 22, 27, 39, 44, 45, 52, 54, 55, 63, 68, 71, 77) described 8 weeks interventions. Exceptions were the Llewellyn-Jones et al. (26) and the Leontjevas et al. (66) studies, which included an assessment after 9.5 and 20 months, respectively. Usually, treatment sessions had a weekly frequency, higher in some studies (2/week) (38, 39, 44), (3/week) (61, 65) and lasted 30 to 60 min (21, 27, 41, 45, 47, 48, 53, 57, 59, 77).

As far as treatment delivery is concerned, interventions were administrated by external experts (23, 26–30, 33, 34, 36, 37, 39, 41, 43–47, 50–52, 54, 55, 57, 59, 60, 62–65, 68, 70–74) or trained staff (31), and in many cases by experts, together with the involvement of staff members (21, 22, 32, 38, 40, 48, 49, 53, 56, 66, 67, 69, 77). Apparently, the cooperation/involvement of an external expert had no impact on treatment effectiveness. In some cases, it was not specified whether treatment was delivered by an external expert or trained member of the staff (25, 42, 76).

Tables 1–**7** summarize the main features of the studies included in the review process: sample (Number–intervention group IG control group CG, Age, Gender W, Drop Out rate), study design, residential type, measures for dementia, measures for depression, intervention (-type, -individual or group setting, -professionals involved, -monotherapy or augmentation), duration and timing of treatment, main findings and quality rate (Jadad and NOS). We grouped studies according to intervention type as follows: horticulture/gardening, pets, physical exercise, psychoeducative/rehabilitation interventions, psychotherapy, reminiscence and story sharing, miscellaneous).

**Table 1:** Horticulture and gardening studies (n 3 articles).

**References**	**Sample Intervention Group IG, Control Group CG (Number, Age, Gender—Woman W, Dropout rate -DR)**	**Study Design**	**Residential type and Country**	**Measures for dementia**	**Measures for depression**	**Intervention (-type, -individual or group setting, -professionist, -monotherapy or augmentation)**	**Duration and timing of treatment**	**Main findings**	**Quality rate (JADAD/NOS)**
Chu et al. (21)	Patients: 150 IG: 75 CG: 75 Mean age: IG 79.2 CG 77.9 W: IG 66.7% CG 58.7% DR: 0%	RCT	Nursing homes; Taiwan	MMSE	GDS-15	-horticultural activity -group -not specified who is the leader -not specified if other therapies	8 week horticultural activity 1.5–2 h sessions weekly	A reduction in depressive symptoms was observed in IG, while an increase was observed in CG	JADAD 6.5
Lai et al. (22)	Patients: 111 IG: 56 CG: 55 Mean age: all 84.6 W: all 65,6% DR: 14%	RCT	Nursing homes; Hong Kong	Abbreviated Mental Test	GDS-15	horticultural therapy -group setting -registered horticultural therapist -not specified if other therapies	Weekly 60 min session for 8 consecutive weeks	Significant interaction effect between group and time was observed only on the happiness scale.	JADAD 6.5
Park et al. (23)	Patients: 50 IG: 24 CG: 26 Mean age: IG: 79.4 CG: 84.5 W: all 100% DR: 0%	QED	Nursing home; Korea	MMSE	GDS-15	-gardening intervention -group setting -horticultural therapist -monotherapy/augmentation: not specified	15 session 50 min/session	Depression scores in IG did not change, while CG exhibited an increase in depression scores.	NOS 6

### Synthesized Findings

#### Horticulture and Gardening

Three studies on horticulture/gardening were included in our systematic review (Table 1). These focused both on physical well-being and on the effects of increased social interaction involved in the activity itself. One study (21) described a significant decrease (i.e., an improvement) in the mean scores for depression in the experimental group, and the opposite in the control group (SMD −15.21; CI 95% −16.98 to −13.44). The randomized controlled trial by Lai et al. (22) highlighted a significant effect of the intervention from a subjective perspective (increased subjective happiness), but not from an objective one (GDS score). Last, the study by Park et al. (23) failed to observe any change in depression scores in elderly women living in Korean nursing homes, and a worsening of depression scores (from normal scores to moderate depression) in the control group during the observation period. SMDs, CI 95% are reported in Supplementary Table 1 and Supplementary Figure 1.

#### Pet Therapy

Four studies on pet therapy were included in our systematic review (Table 2); pets were dogs in 3 of them and cats in one (38). Three of these studies (38–40) showed an improvement of elderly patients' depressive symptoms, while no significant change in the GDS score was found in Phelps' sample (5 patients) (41). With more detail, Ambrosi et al. (40) highlighted that dog assisted therapy could reduce depressive symptoms in elderly people by facilitation of social interactions and consequent elicitation of a positive emotional response (SMD −1.39; CI 95% −2.22 to −0.56). SMDs, CI 95% and further details are reported in Supplementary Table 2 and Supplementary Figure 2.

**Table 2:** Pet therapy studies (n 4 articles).

**References**	**Sample Intervention Group IG, Control Group CG (Number, Age, Gender—Woman W, Dropout rate -DR)**	**Study Design**	**Residential type**	**Measures for dementia**	**Measures for depression**	**Intervention (-type, -individual or group setting, -professionist, -monotherapy or augmentation)**	**Duration and timing of treatment**	**Main findings**	**Quality rate (JADAD/NOS)**
Ambrosi et al. (40)	Patients: 31 IG: 17 CG: 14 Mean age: IG: 86.2 CG: 87.1 W: IG: 88% CG: 100% DR: 3.2%	RCT	Long Term Care facility; Italy	MMSE	GDS-15	30 min sessions of dog-assisted therapy (DAT) with a dog handler, recording verbal and non-verbal interactions with the dog	30 min sessions, for 10 weeks	A greater reduction in depressive symptoms was observed in IG compared to CG	JADAD 5
Sollami et al. (39)	Patients: 28 IG: 14 CG: 14 Mean age IG: 85.07 CG: 84.91 W: not assessed DR: 0%	QED	Nursing home; Italy	MMSE	GDS-15	-pet therapy caregiving activities; Participants were taught the correct method of physically interacting with the dog, aimed to stimulate the manipulation -operator	16 AAI meetings: 2 times a week, with 1 h sessions	Quality of life and depression improved in IG	NOS 5
Stasi et al. (38)	Patients: 28 IG: 14 CG: 14 Mean age: IG: 85 CG: 86,4 W: not assessed DR: 0%	QED	Nursing home, Italy	MMSE	GDS-15	-pet therapy (cat) -supervisor, nurse	3/week sessions of almost 1 h visit for 6 weeks	A reduction in depressive symptoms was observed in IG	NOS 4
Phelps et al. (41)	Patients: 5 Mean age: 84.5 W: 60% DR: 0%	NE	Nursing home, USA	MMSE	GDS-15 and Geriatric PANAS	-dog visit -group of 2 -nurse -3 out of 5 patients in augmentation	1 visit/ week for 6 weeks, for at least 5 min and up to 10 min	None of the five residents showed improvement in GDS scores	NOS 4

#### Physical Exercise

Nine studies dealt with physical exercise, including different approaches and exercise programmes (Table 3).

**Table 3:** Physical exercise studies (n 9 articles).

**References**	**Sample Intervention Group IG, Control Group CG (Number, Age, Gender—Woman W, Dropout rate -DR)**	**Study Design**	**Residential type**	**Measures for dementia**	**Measures for depression**	**Intervention (-type, -individual or group setting, -professionist, -monotherapy or augmentation)**	**Duration and timing of treatment**	**Main findings**	**Quality rate (JADAD/NOS)**
De Carvalho Bastone and Filho (25)	Patients: 40 IG: 19 CG: 18 Mean age: IG: 76.78 CG: 80.25 W: IG: 73.7% CG: 77.8% DR: 7.5%	QED	Nursing home, Brazil	MMSE	GDS-30	1 h exercise session in group setting (mobility, strengthening, closed-kinetic chain, walk and relaxation)	1 h exercise session, once a week for 6 months	A reduction in depressive symptoms was observed in IG.	NOS 6
Brown et al. (42)	Patients: 154 3 groups: -GE (general group-based exercise): 82 -FR (flexibility exercise and relaxation technique): 34 -NEC (no exercise control): 38 Mean age: GE: 79.5 FR: 81.5 NEC: 78.1 W: all 87.7% DR: GE: 20% FR 24% NEC 11%	RCT	Intermediate care and self-care retirement village, Australia	MMSE	GDS-15; PANAS	1 h exercise classes twice a week for 6 months, with experienced instructors	6 months intervention	Baseline GDS and PANAS scores were worse in FR than in GE group. A decrease of depression was observed in GE and FR groups, in patients with initially high GDS scores	JADAD 5
Coelho et al. (65)	Patients: 42 2 Groups: Music and Movement (MMG): 20 Mean age: 80.65 Cognitive training without music (CTG): 19 Mean age: 83.68 y W: all 83% DR: 7%	QED	Nursing homes; Portugal	MMSE, MPCR	GDS-27	Music and Movement Group Musical selection based on the participants' preferences Sensory-motor activity	The intervention was carried out for 4 months, 3/week, 60 min (16 weeks)	A reduction in depressive symptoms was observed in the both groups (higher in MMG)	NOS 7
Chao et al. (44)	Patients: 7 Mean age: 86 W: 71% DR: 0%	NE	Assisted living facility; USA	MMSE	GDS-15	-stay active, healthy aging self-efficacy theory based program -group of two researchers -not specified if monotherapy or augmentation	2 session/week 8 weeks (16 exercise session)	No statistically significant results	NOS 4
Chen et al. (61)	Patients: 69 IG: 38 CG: 31 Mean age 75.40 W 52.70% DR: 19.2%	QED	Assisted living facilities, Taiwan	MMSE	Taiwanese Depression Questionnaire	-yoga -group setting -certified instructors yoga -not specified if other therapies	Three times a week for 6 months	A reduction in depressive symptoms was observed in IG.	NOS 6
Chen et al. (60)	Patients: 127 IG: 64 CG: 63 Mean age: 79.15 W: all 49.1% DR: 9.5%	RCT	Nursing homes, Taiwan	SPMSQ	TDQ	-wheelchair -bound senior elastic band exercise program -group -trained and certified instructors -not specified if other therapies	Three times per week 40 min per session for 6 months. Data were examined at baseline, at 3 months, and at the end of the 6 month study	A reduction in depressive symptoms was observed in IG, while an increase was observed in CG	JADAD 5
Roswiyani et al. (45)	Patients: 299 Art group A: 63 Qigong group Q: 67 Art and Qigong AQ: 72 C: 65 Mean age: A: 74,19 Q: 71.90 AQ: 74.83 C: 74.31 W: A 61.9% Q: 70.1% AQ: 75% C: 66.2% DR: 6%	RCT	Nursing homes, Indonesia	MMSE	GDS-15 BDI-II	-(1) a group with Qigong and art activities as an, integrated intervention, (2) a group with art activities, (3) a group with Qigong, and (4) a control group that gets no intervention	Eight weeks (16 sessions), lasting 90 min each	In the art group it was observed a significant positive effect on well-being and a reduction in depressive symptoms	JADAD 5
Vankova et al. (43)	Patients: 167 IG: 79 CG: 83 Mean age IG: 83.38 CG: 82.85 W: IG: 91.6% CG: 92.4% DR: 0%	RCT	Nursing home, Czech Republic	MMSE	GDS-15	-Dance therapy -group -dance instructor -augmentation to antidepressant therapy for 29 patients	Once a week for 1 h for 3 months	A reduction in depressive symptoms was observed in IG	JADAD 4
Winningham et al. (70)	Patients: 72 IG: 25 CG: 17 Mean age: all 81.7 W: not assessed DR: 41.67%	QED	Assisted living facilities, USA	MMSE	GDS	MemAerobics Mixed individual and group session Trained instructor	3 times/week the MemAerobics group engaged in fun and social activities designed to stimulate cognitive functioning	A reduction in depressive symptoms was observed in IG	NOS 5

As reported in Supplementary Table 3 and Supplementary Figure 3, Coelho et al. (65) (SMD −0.90; CI 95% −1.56 to −0.24), De Carvalho Bastone and Filho (25) (SMD −0.82; CI 95% −1.49 to −0.14), Brown et al. (IG1 vs. IG2) (42) (SMD −0.77; CI 95% −1.18 to −0.36 in GDS score, SMD −0.56; CI 95% −0.97 to −0.15 in PANAS-P score), Chen et al. (60) at 3 month follow-up (SMD −0.75; CI 95% −1.11 to −0.39), Chen et al. at 6 month follow-up (SMD −0.68; CI 95% −1.04 to −0.32) (60), Chen et al. at 6 month follow-up (SMD −0.63; CI 95% −1.18 to −0.09) (61) showed a significant improvement in depression after exercise interventions. The intervention used by those studies were: wheelchair-bound senior elastic band (60), music plus movement (65), Yoga (61), mobility, strengthening, closed-kinetic chain, walk, and relaxation (25).

On the other hand, the effectiveness of Wii Fit (44) or Qui gong exercise (45) remain uncertain on participants' level of depression.

#### Psychoeducational and Rehabilitation Interventions

Fifteen studies on psychoeducational and rehabilitation interventions were included in our systematic review (Table 4). The interventions in this group were performed in individual or group setting, either self-managed after training or managed by experienced staff. Among the heterogeneous interventions described, several authors reported a positive effect on depressive symptoms (26–33, 46, 62, 66, 71, 72). The types of psychoeducational/rehabilitation interventions assessed included: 8 week sessions of writing group (45); training, education and activity programmes for residents as well as for physicians and carers (26); a mixed intervention including different activities with a recreation therapist (27); an individualized psychosocial intervention carried out by a volunteer and a nurse (28); mentoring English conversational activity (29); self-worth therapy managed by a trained mental health nurse (30); a program of pleasant event activities (31); videoconference interaction with relatives (32); “friendly visits” problem-solving treatment (72); a specific multidisciplinary care program (66); a Socially Supportive Activities Program intervention (62); facility adaptation promotion program (46); nurse-led sleep program (33).

**Table 4:** Psychoeducative/Rehabilitation Interventions studies (n 15 articles).

**References**	**Sample Intervention Group IG, Control Group CG (Number, Age, Gender—Woman W, Dropout rate -DR)**	**Study Design**	**Residential type**	**Measures for dementia**	**Measures for depression**	**Intervention (-type, -individual or group setting, -professionist, -monotherapy or augmentation)**	**Duration and timing of treatment**	**Main findings**	**Quality rate (JADAD/NOS)**
Cernin et al. (31)	Patients: 25 IG: 8 CG: 7 Mean age: IG: 82.9 CG: 83.7 W: IG: 100% CG: 71.4% DR: 40%	RCT	Assisted living community, USA	BTOT	GDS-30	-pleasant event activities mutually agreed upon with residents -individual setting -staff -not specified if other therapies	30 min sessions, 3 sessions per week for 3 months	Intervention may result in improved affective functioning	JADAD 2
Cesetti et al. (47)	Patients: 30 IG: 20 CG: 10 Mean age: IG: 79.35 CG: 76.50 W: IG: 75% CG: 60% DR: 0%	QED	Nursing home, Italy	MMSE	GDS-15	-positive narrative group intervention -group setting -clinical psychologist -not specified if other therapies	2 h sessions, 4 weekly sessions	No significant change in GDS score in any group. An improvement of well-being and sleep quality was observed in IG	NOS 7
Dolu et al. (33)	Patients: 52 IG: 26 CG: 26 Mean age: IG: 80.69 CG: 78.92 W: IG: 57.7% CG: 46.2% DR: 7.7%	QED	Nursing home, Turkey	SMMTE	GDS-30	- nurse-led sleep programme, and motivational interview -individual setting -the author -not specified if other therapies	1 h sessions, once a week for 4 week	A reduction in depressive symptoms was observed in IG	NOS 6
Dozeman et al. (67)	Patients: 129 IG: 67 CG: 62 Mean age: IG: 83.7 CG: 84.2 W: IG: 68.7% CG: 80.6% DR: IG: 23.8% CG: 9.7%	RCT	Residential home and nursing home; Netherlands	MMSE	CES-D	-guided self-help course, with a standardized treatment schedule -individual setting -instructed staff an volunteers (coaches) -augmentation to activity program on a daily basis and usual medications	2-5 support visits by the coach.	A (non-statistically significant) reduction in depressive symptoms was observed IG	JADAD 6.5
Hsu et al. (62)	Patients: 79 IG: 40 CG: 39 Mean age: IG: 79.57 CG: 79.52 W: IG: 74.3% CG: 48.5% DR: IG: 12.5% CG: 15.4%	RCT	Long-term care facility, Taiwan	SPMSQ	GDS-15; AERS	-Socially Supportive Activities Program (SSAP) -group setting -trained researcher -not specified if other therapies	60 min group Activities, 10 weekly sessions	Significant reduction in depressive symptoms (GDS) and significantly better mood (AERS) after intervention	JADAD 5
Leontjevas et al. (66)	Dementia Units Patients: 403 Mean age: 83.4 W: 70.1% DR: 46% Somatic Units Patients: 390 Mean age: 77.4 y W: 64.2% DR: 42%	RCT	Nursing home, Netherlands	MMSE	CSDD GDS-8	-Act in Case of Depression (multidisciplinary care program-AiD) -individual and group setting -nursing staff, activity therapists, psychologists and physicians -monotherapy	Groups start the intervention at 4 month intervals (total period: from 4 to 20 months)	In somatic units, but not in dementia units, intervention reduced the prevalence of depression	JADAD 7
Llewellyn Jones et al. (26)	Patients: 220 IG: 109 CG: 111 Mean age: IG: 84.9 CG: 83.8 W: IG: 83% CG: 86% DR: IG: 21.1% CG: 25.2%	RCT	Nursing home, Australia	MMSE and BOMCT	GDS-30	-training, education and activity programmes for GP, carers and residents in detection and management of depression -individual and group setting -GP -augmentation to antidepressant for <20% patient	Assessment at baseline and 9.5 month	A reduction in depressive symptoms was observed in IG. Training, education and activity programmes enhance clinical skills of GP and improve patients' symptoms	JADAD 7
McCurren et al. (28)	Patients: 85 IG: 44 CG: 41 Mean age: IG: 83.84 CG: 85.12 W: IG: 86% CG: 78% DR: IG: 22.7% CG: 34.1%	RCT	Nursing home, USA	MMSE	GDS-30	-intervention strategy for depression -individual setting -peer counselor volunteers trained and supervised by a master's-prepared geropsychiatric nurse -50% of the treatment group and 17% of the control group were on antidepressants	24 weeks, visits 2 times per week by the volunteer, and weekly visits by the nurse	A reduction in depressive symptoms was observed in IG. The use of antidepressants was not significant	JADAD 5
Reinhardt et al. (72)	Patients: 37 IG: 21 CG: 16 Mean age: IG: 75.2 CG: 75.9 W: IG: 52.4% CG: 50% DR: IG: 66.7% CG: 12.5%	RCT	Nursing home, USA	MMSE	PHQ- 9, PROMIS depression scale, SCID, HAM-D	-PST and “friendly visits” (social contact group) -no specified -PST therapist, interns (for social contact group) -not specified if other therapies	PST: 1 h sessions, 6 session; social contact comparison: 6 “friendly” visits	A reduction in depressive symptoms was observed in IG	JADAD 4
Rosen et al. (27)	Patients: 31 IG: 11 CG: 11 Mean age: all 78.7 W: all 64.5% DR: 29%	NE	Nursing home, USA	MMSE	HAM-D GDS-30	-control-relevant psychosocial intervention (different activities with recreation therapist). -group setting -licensed recreation therapist -not specified if other therapies	1 2 h sessions, twice per day for 5 days each week, for 8 weeks	Intervention enhanced socialization and had a positive impact on almost half of the patients with depression	NOS 4
Sok et al. (46)	Patients: 73 IG: 36 CG: 37 Mean age: IG: 72.06 CG: 73.02 W: IG: 61.1% CG: 64.9% DR: 0%	QED	Nursing home, Korea	MMSE	GDS-15	-Facility adaptation promotion program (group education and group discussion) -group setting -researcher -not specified if other therapies	60 min sessions, once a week for 10 weeks	A reduction in depressive symptoms was observed in IG, furthermore, it improved self-esteem, relationships, life satisfaction and adaptation to facility	NOS 6
Supiano et al. (71)	Patients: 116 IG: 62 CG: 54 Mean age: IG: 82.3 CG: 84.7 W: IG: 79.2% CG: 70.6% DR: 41.9%	QED	Nursing home, USA	MMSE	DS	-Writing groups -group setting -instructor with a master's degree in counseling and human Development; trained volunteers -monotherapy	Once a week for 8 weeks	A reduction in depressive symptoms was observed in IG.	NOS 5
Tsai et al. (30)	Patients: 111 IG: 55 CG: 56 Mean age: IG: 78.5 CG: 76.3 W: IG: 61.3% CG: 65.6% DR: IG: 43.6% CG: 42.9%	QED	Nursing home, Taiwan	MMSE	GDS-30	-self-worth therapy -individual setting -Master's-prepared mental health nurse -in augmentation to antidepressants, if previously prescribed	30 min sessions, once a week for 4 weeks	A reduction in depressive symptoms was observed in IG	NOS 5
Tsai and Tsai (32)	Patients: 90 IG: 40 CG: 50 Mean age: IG: 73.82 CG: 79.26 W: IG: 55% CG: 60% DR: IG: 44% CG: 33%	QED	Nursing home, Taiwan	MMSE	GDS-30	-Videoconference interaction with their family members -individual setting -trained research assistant, then trained nursing home staff -in augmentation to usual family visits	Once a week for 3 months	A reduction in depressive symptoms was observed in IG at 3, 6, and 12 months after the intervention	NOS 7
Yuen et al. (29)	Patients: 28 IG: 15 CG: 13 Mean age: IG: 83.0 CG: 83.9 W: IG: 80% CG: 62 % DR: IG: 13.3% CG: 0%	RCT	Long term facility, USA	MMSE	GDS-30 LSI-A	-Mentoring English conversational skills for English-as-a-second-language (ESL) student -individual -English-language teachers/ESL -not specified if other therapies	1 h sessions, twice per week for 12 weeks	Participants had significantly higher levels of well-being, lasting up to 3 month follow-up	JADAD 4

Sok (SMD −2.73; CI 95% −3.37 to −2.08) (46); Hsu and Wang (SMD −1.51 CI 95% −2.02 to −1.01) (52), Dolu and Nahcivan (8 weeks follow up SMD −1.34; CI 95% −1.98 to −0.70; 12 weeks follow up SMD −1.20; CI 95% −1.85 to −0.56) (33) showed larger positive effect.

Effect sizes for each study arm are reported in Supplementary Table 4 and Supplementary Figure 4.

Two out of these 15 studies failed to find significant changes in depressive symptoms: the study by Dozeman et al. (67), about a guided self-help course, and that by Cesetti et al. (47), dealing with a narrative group intervention.

#### Psychotherapy

Three studies on cognitive-behavior psychotherapy were included in the review (Table 5). Two of them supported its effectiveness on depression (34, 48), while one did not (77).

**Table 5:** Psychotherapy studies (n 3 articles).

**References**	**Sample Intervention Group IG, Control Group CG (Number, Age, Gender—Woman W, Dropout rate -DR)**	**Study Design**	**Residential type**	**Measures for dementia**	**Measures for depression**	**Intervention (-type, -individual or group setting, -professionist, -monotherapy or augmentation)**	**Duration and timing of treatment**	**Main findings**	**Quality rate (JADAD/NOS)**
Blair et al. (77)	Patients: 6 Mean age: 88.2 W: not assessed DR: 16.7%	NE	Low-level residential care facility, Australia	MMSE	BDI-II; BHS; DASS 21	-cognitive behavior therapy (CBT) -group setting -researchers -not specified if other therapies	2 h sessions, once a week for 8 weeks	Two participants improved on the BDI, 1 worsened slightly	NOS 2
Hyer et al. (48)	Patients: 25 IG: 13 CG: 12 Mean age: IG: 78 CG: 81 W: all 16% DR: 0%	QED	Veteran's nursing home, USA	MMSE	GDS-15	-cognitive behavioral therapy (GIST) -group and individual setting -therapist and selected staff -not specified if other therapies	75 to 90 -min sessions,15 sessions on 2 weeks	A reduction in depressive symptoms was observed in IG	NOS 6
Konnert et al. (34)	Patients: 64 IG: 35 CG: 29 Mean age: 81,1 W: all 77% DR: IG: 42.8% CG: 20.7%	RCT	Non-profit nursing home, Canada	MMSE	GDS-30	-adaptation of the coping with Stress program (cognitive–behavioral treatment/CBT)-group setting -doctoral-level -clinical psychology students -not specified if other therapies	13 sessions over 7 weeks	A reduction in depressive symptoms was observed in IG	JADAD 5

SMDs for each study arm are reported in Supplementary Table 5 and Supplementary Figure 5.

#### Reminiscence and Story Sharing

Our review included 14 studies on reminiscence and story sharing (Table 6). Reminiscence, life review and story sharing yielded significant results on depression in 12 studies (35–37, 49–54, 63, 73, 74) while two recent RCTs (68, 75) failed to find a significant effect of the reminiscence therapy intervention on depressive symptoms.

**Table 6:** Reminiscence and Story Sharing studies (n 14 articles).

**References**	**Sample Intervention Group IG, Control Group CG (Number, Age, Gender—Woman W, Dropout rate -DR)**	**Study Design**	**Residential type**	**Measures for dementia**	**Measures for depression**	**Intervention (-type, -individual or group setting, -professionist, -monotherapy or augmentation)**	**Duration and timing of treatment**	**Main findings**	**Quality rate (JADAD/NOS)**
Chao et al. (49)	Patients: 24 IG: 12 CG: 12 Mean age: IG: 79.58 CG: 76.92 W: IG: 33.3% CG: 33.3% Drop out: IG: 16.7% CG: 33.3%	QED	Nursing home, Taiwan	MMSE	GDS-15	-reminiscence group therapy -group setting -college -level instructor who specialized in psychiatric nursing -not specified if other therapies	1 h sessions, 9 sessions once a week	A reduction in depressive symptoms was observed in IG	NOS 6
Chiang et al. (63)	Patients: 130 IG: 65 CG: 65 Mean age: IG: 77.42 CG: 77.06 W: IG: 0% CG: 0% DR: IG: 31% CG: 28%	RCT	Nursing home, Taiwan	MMSE	CES-D	-reminiscence group therapy -group setting -master's prepared student inmental health nursing -not specified if other therapies	90 min sessions, once a week for 8 weeks	A reduction in depressive symptoms was observed in IG	JADAD 5
Chuang et al. (50)	Patients: 70 IG: 35 CG: 35 Mean age IG: 84.38 CG: 84.23 W: IG: 72.4% CG: 61.3% DR: IG: 17.1% CG: 11.4%	RCT	Long-term care facility, Taiwan	SPMSQ	GDS-15	-Story-Centered Care Intervention Program -group setting -trained researcher -not taking any antidepressants	60–90 min sessions, once a week for 4 weeks	A reduction in depressive symptoms was observed in IG	JADAD 6.5
Chueh et al. (36)	Patients: 21 IG: 11 CG: 10 Mean age: all 82 W: 0% DR: IG: 36% CG: 40%	QED	Veterans' nursing home, Taiwan	SPMSQ	GDS-30	-reminiscence therapy -group setting -therapist with extensive experience and trainingin group counseling and reminiscence therapy -not specified if other therapies	60 min sessions, 2 times a week for 4 weeks	A reduction in depressive symptoms was observed in IG	NOS 5
Haight et al. (73)	Patients: 52 IG: 29 CG: 23 Mean age: 79.6 W: 69% DR: 0%	RCT	Nursing home, USA	MSQ	NIMH Diagnostic Interview Schedule, BDI, HS, BSI	-Structured Life review -individual setting -therapeutic listener -not specified if monotherapy or augmentation	Missing information	A trend toward continued and significant improvement over time in measures of depression, life satisfaction and self-esteem was observed in those who received the life review intervention	JADAD 3
Hamzehzadeh et al. (37)	Patients: 27 IG: 15 CG: 12 Mean age: IG: 77.9 CG: 74.1 W: 100% DR: IG: 33.3% CG: 8.3%	RCT	Nursing home, Iran	MMSE	GDS-30	-Reminiscence therapy -group setting -examiner -not specified if monotherapy or augmentation	1 h sessions, twice a week for 4 week	A reduction in depressive symptoms, and an improvement of optimism were observed in IG	JADAD 4
Hsu and Wang (52)	Patients: 45 IG: 21 CG: 24 Mean age IG: 77.9 CG: 77.9 W: IG: 47.8% CG: 50% DR: 0%	QED	Long-term care facility, Taiwan	MMSE	GDS-15	- reminiscence sessions -group setting -master's-prepared -geriatric nurse specialists -not specified if other therapies	60 min sessions, 6 to 8 sessions, once a week over 2 months	A reduction in depressive symptoms and an improvement of behavioral competence were observed in IG	NOS 7
Jones (35)	Patients: 30 IG: 15 CG: 15 Mean age: IG: 82.7 CG: 81 W: all 100% DR: 0%	QED	Long term care facility, USA	MMSE	GDS-30	-Nursing Intervention Classification Reminiscence therapy -group setting -professional (master nurse) - not specified if other therapies	45 min sessions, twice a week for 3 weeks	A reduction in depressive symptoms was observed in IG	NOS 6
Karimi et al. (53)	Patients: 39 Integrative Group: 10 Instrumental group: 9 CG: 10 Mean age: 70.5 W: Integrative group: 60% Instrumental group: 77.8% CG: 70% DR: 26%	RCT	Nursing Home, Iran	MMSE	GDS-15	-integrative reminiscence intervention and instrumental reminescence intervention -group setting -master's level therapist -in augmentation to antidepressants, if previously prescribed	6 weekly sessions of 90 min	A reduction in depressive symptoms was observed in the integrative reminiscence group	JADAD 5
Lan et al. (51)	Patients: 62 IG: 31 CG: 31 Mean age: IG: 83.06 CG: 82.9 W: IG: 64.52% CG: 61.29% DR: IG: 12.9% CG: 9.7%	RCT	Nursing home, China	SPSMQ	GDS-15	-Life review -group setting -nurse trained infacilitating the life review process -none of the participants were using any antidepressant medication	6 weekly sessions	A reduction in depressive symptoms was observed in IG, which potentially improved self-esteem and meaning in life	JADAD 5
Meléndez-Moral et al. (54)	Patients: 34 IG: 17 CG: 17 Mean age IG: 79.78 CG: 79.75 W: IG: 83.3% CG: 56% DR: 0%	RCT	Long term care facility, Spain	MMSE	MINI, GDS-8 and GDS-15	-reminiscence therapy -group setting -held by psychologist -monotherapy	8 sessions of 60 min	A reduction in depressive symptoms was observed in IG; an increase was observed in CG	JADAD 3.5
Sullivan et al. (75)	Patients: 100 IG: 48 CG: 52 Mean age: IG: 81.5 CG: 81.5 W: IG: 62.5% CG: 63.4% DR: IG: 14.6% CG: 0%	RCT	Long term facility, USA	Mini-Cog	DI	-Story Sharing -group setting -investigator (not specified) - not specified if other therapies	30 min sessions, twice a week for 3 weeks	Intervention did not improve depressive symptoms	JADAD 5
Westerhof et al. (68)	Patients: 81 IG: 42 CG: 39 Mean age: all 84.2 W: all 62% DR: IG: 27.9% CG: 28.2%	RCT	Residential Care, Netherlands	MMSE	GDS-8	-”Precious memory” (autobiographical memory intervention)-individual setting -Trained volunteer -not specified if other therapies	45 min sessions, weekly or biweekly for 8 weeks maximum	A reduction in depressive symptoms was observed in both groups	JADAD 5
Zauszniewski et al. (74)	Patients: 43 Mean age: 84 W: 79.1% DR: 25.58%	QED	Retirement community, USA	SPMSQ	ESC	-focused reflection reminiscence program -group setting -advanced practice psychiatric nurse -not specified if other therapies	2 h sessions, once a week for 6 weeks	A reduction in depressive symptoms was observed IG	NOS 6

SMDs are reported in Supplementary Table 6 and Supplementary Figure 6.

#### Miscellaneous

In the miscellaneous group we included 8 studies about different and heterogeneous approaches which did not seem to fit any of the above-described categories (Table 7).

**Table 7:** Miscellaneous articles (n 8 articles).

**References**	**Sample Intervention Group IG, Control Group CG (Number, Age, Gender—Woman W, Dropout rate -DR)**	**Study Design**	**Residential type**	**Measures for dementia**	**Measures for depression**	**Intervention (-type, -individual or group setting, -professionist, -monotherapy or augmentation)**	**Duration and timing of treatment**	**Main findings**	**Quality rate (JADAD/NOS)**
Biasutti and Mangiacotti (59)	Patients: 45 IG: 20 CG: 25 Mean age: IG: 83.79 CG: 83.42 W: IG: 68% CG: 58% DR: 13%	RCT	Residential center, Italy	MMSE	GDS-15	Music therapy and Music training, supervised by a music psychologist.Gymnastic activities supervised by an expert physiotherapist	70 min sessions, twice a week, of intensive music training program Non-music experimental intervention in the control group: 45 min gymnastic activities, twice a week	A reduction in depressive symptoms was observed in IG In the group with MMSE >23, no significant improvement in the GDS	JADAD 5
Bosmans et al. (69)	Patients 185 IG: 93 CG: 92 Mean age: IG: 84 CG: 84 W: IG: 72% CG: 74% DR: IG: 38% CG: 22%	RCT	Residential homes, Netherlands	MMSE	CES-D	-Stepped care prevention programme -individual and group setting -staff and trainedand supervised mental health nurses -prescription of antidepressants or reference tomental health specialist, if CES-D ≥16	1 month of watchful waiting and then cycles of 3 months of activity schedule	This intervention was not cost-effective and unsuccessful in preventing depression	JADAD 5
Chang et al. (57)	Patients: 20 IG: 10 CG: 10 Mean age 84 W 30% DR: 20%	RCT	Nursing home, Taiwan	MMSE	GDS-15	- transcutaneous electrical acupoint stimulation -individual -nurses -not specified if other therapies	15 min, 5 times per week for 1 month	A reduction in depressive symptoms was observed in IG	JADAD 5
Chiu et al. (58)	Patients: 57 ICT-communication: 19 ICT-entertainment: 18 CG: 17 Mean age: ICT-communication: 72.9 ICT-entertainment: 72.9 CG: 73.2 W: ICT-communication: 42% ICT-entertainment: 50% CG: 59% DR: 5%	RCT	Assisted-living facilities, Taiwan	SPMSQ	CES-D	ICT-communication (using app for instant communications on electronic devices) group ICT-entertainment (using YouTube) Usual care group -group -gerontologist -not specified if other therapies	Once a week for 12 weeks Each session lasted ~90 min	A reduction in depressive symptoms was observed in both IG	JADAD 3
Han et al. (56)	Patients: 42 IG: 19 CG: 18 Mean age: IG: 63,2% <80 CG: 55.6% <80 W: IG: 42.1% CG: 55.6% DR: 11%	QED	Nursing care hospitals, Korea	MMSE	GDS-15	Han's laugh protocol, reviewed by professionists(1 nursing faculty, 3 nurses from nursing homes, 2 instructors)	40 min twice a week for a total of eight sessions held in the patients' lounge = 1 month	A reduction in depressive symptoms was observed in IG	NOS 5
Rajagopal et al. (76)	Patients: 22 Prayer Wheel group: 14 Prayer Wheel individual: 8 Mean age 83.1 W: Prayer Wheel group: 78% Prayer Wheel individual: 87,5% DR: 0%	QED	Continuing care retirement communities; USA	MMSE	CES-D	-Prayer Wheel group cohort -PrayerWheel Individual cohort	Six weeks	A reduction in depressive symptoms was observed in group cohort	NOS 6
Tai et al. (64)	Patients: 60 IG: 41 CG: 19 Mean age: IG: 80.49 CG: 81.68 W IG: 53.7% CG: 52.6% DR: 25%	QED	Senior citizen apartments, Taiwan	MMSE	GDS-SF	- Music therapy with Buddhist hymns -individual setting -music therapist augmentation to standard care	30 min twice a day from Monday to Friday for 4 months	A reduction in depressive symptoms was observed in both groups	NOS 6
Wang et al. (55)	Patients: 76 IG: 35 CG: 41 Mean age 80.5 W: IG: 60% CG: 63.4% DR: 8.4%	RCT	Assisted-living facilities and nursing home, China	SPMSQ or MMSE	GDS-15	Gerotranscendence group -group -Master's prepared psychiatric nurse	Exp group: 60 min a week, for 8 weeks. Control group: 30 min weekly general chatting	A (non-statistically significant) reduction in depressive symptoms was observed IG	JADAD 6.5

These miscellaneous interventions were: a pilot study regarding wheel prayer group activity (76); gero-transcendence, a particular form of transcendence intervention (55); Han et al.'s laugh protocol (56), a 1 month intervention program of trans-cutaneous electrical acupoint stimulation (TEAS) (57); a group activity based on ICT (using app for instant communications on electronic devices) Chiu and Wu (58); music therapy (59, 64).

One study focused on the cost-effectiveness of a stepped-care program to prevent depression and anxiety in elderly homes (69).

All these miscellaneous interventions yielded some results, except for the one by Biasutti and Mangiacotti (59) and that by Bosmans et al. (69), which was neither cost-effective nor successful in preventing depression.

SMDs are reported in Supplementary Table 7 and Supplementary Figure 7.

#### Study Quality Assessment

The overall quality of RCTs was assessed with the Jadad; QED and NE studies were assessed using NOS and resulted quite poor (some studies involving small samples and/or with a poor methodological approach). The results regarding studies quality were consistent with those reported by the previous review by Simning and Simons (9) which highlighted an overall lack of quality of the studies in the field (using the Jadad scale).

## Discussion

### Summary of Main Findings

The aim of this systematic review was to assess interventions focused on depression in elderly with no or mild cognitive impairment living in LTCFs. To our knowledge, three previous literature reviews (8, 9, 78) focused on depression among older people living in LCTFs and Nursing Homes and were performed with an approach which was similar to our own (at least as far as inclusion criteria and methods are concerned), albeit not totally overlapping. Simning and Simons (9) and Yoon et al. (8) included studies on both pharmacological and non-pharmacological interventions (psychotherapeutic, recreational, psychosocial and pharmacologic or other biologic interventions). Davison et al. (78) focused on psychological treatments in older adults living in LTCFs, but included those with a diagnosis of dementia or other comorbidities as well. Furthermore, they excluded rehabilitation and exercise-related activities and limited their review to reminiscence and life review interventions. Hence these review works eventually included different types of studies.

Studies included in the current review were highly heterogeneous as regards type, intervention methodology (duration, sessions number and frequency, setting -individual or group), number of participants, eligibility criteria, professional performing the intervention (members of the LTCF internal staff or external expert), type of residential facility.

Furthermore, as already reported by previous reviews (9, 78), regrettably the studies available in the literature define depression criteria to a limited degree only. They usually lack a clinical diagnosis on behalf of an experienced psychiatrist; furthermore, most of the studies included in our review did not consider lifetime history of depression, comorbid medical illnesses or concomitant psychopharmacological treatments.

Most studies consider the decrease in depressive symptomatology level (investigated by validated psychometric questionnaires) as study outcome. Nonetheless, the assessment of changes in a depression rating scale score could not provide enough information about real mental health and clinical depression before and after intervention. The assessment of an actual improvement or remission of depression would imply a psychiatric interview and clinical instruments for diagnosis according to DSM or ICD criteria (78); actually, a clinical psychiatric assessment was one of the activities proposed by the stepped care approach in Leontjevas et al. (66).

The overall duration (weeks) of the intervention delivered seemed to have a greater impact on treatment effectiveness than the duration of each single session.

Forty-one percent of the studies considered in our systematic review were performed in the Asian continent; we can hypothesize that the rate of older adults in a given country, its cultural context, the perception of and the approach to aging might play a role from this standpoint. All these issues may eventually lead to differences in the attention paid to different types of non-pharmacological treatment for depressed elderly. For example, horticultures interventions were conducted only in the Asian continent, while most (75%) of the articles dealing with pet therapy come from Italy (38–40). This result can be related to the more holistic and spiritual vision of the Asian culture, characterized by a thorough approach including body, mind and spirit (79) and spirituality (80), which can increase Asian researchers' interest in non-pharmacological therapeutic approaches for elderly's depression.

Summarizing the main findings from the current review, most of the included studies seemed to suggest some kind of effectiveness of the proposed intervention on depression. Through our systematic literature review we were able to identify seven types of intervention, and in each one there are effective interventions with different effect sizes. Nonetheless, variability in sample size and eligibility criteria makes it difficult to compare results from different studies and to draw clear conclusions. Differences in participants' mean age, and even more, the choice to include/exclude soft or mild cognitive impairment, certainly affected the research findings. Due to this kind of heterogeneity a meta-analysis could not be performed; nonetheless, physical exercise, psychotherapy, and reminiscence interventions (see Supplementary Tables 3, 5, 6 and Supplementary Figures 3, 5, 6, respectively), seem to be the most effective interventions.

Furthermore, as described also by Simning and Simons (9), LTCFs constitute a widely heterogeneous context, spanning from low-level dependency to high-level dependency facilities, and hence offering different types of assistance. Regarding the populations involved in the studies we assessed, one more methodological limitation could be participants' selection: in many studies the sample was randomized, while in other studies sampling was largely dependent on type of LTCF, setting, population and residents' willingness to participate to the proposed intervention.

While the Yoon and co-workers' (8) review emphasized the importance of providing individualized treatments, no specific focus on individualization emerged from the articles included in the current review. Another limitation, which is inherent the specific population and setting we investigated, was about the frequency of drop out and the possibility for participants to complete the follow-up. Thus, the results should be considered with caution, in the light of the several limitations which are further detailed below.

### Limitations

Some limitations of the current review should be underscored. We selected articles in English language only, which can potentially exclude relevant results published in other languages. We decided to focus on depressed elderly and excluded studies involving those with a severe cognitive impairment (MMSE score <20), which represent only a portion of the global elderly population. Despite mixed or negative findings in some cases, most studies included in this systematic review reported that the non-pharmacological interventions they assessed seemed effective in the management of depressed elderly in the LTCFs context. Nonetheless, making a clear decision about their actual effectiveness is difficult as data reported are very varied, and the populations involved in the studies are varied as well, and sometimes poorly defined. Regrettably, this fact together with the limitations and heterogeneity of the studies described above, hinder the possibility to draw clear conclusions about the actual effectiveness of the interventions analyzed in the papers (78). Despite the limitations described above, we believe that the current review adds to the existing literature as it tried to focus as much as possible on not severely cognitively impaired depressed elderly, selecting studies involving participants with a MMSE score > 20; furthermore, it proposes a categorization of the types of non-pharmacological treatment offered in the context of LTCFs, and includes an accurate analysis of studies quality and effect size of the results obtained.

## Conclusions

Non-pharmacological interventions for the management and treatment of depression in the elderly population could be an important strategy, as this population could be particularly frail and sensitive with regard to pharmacological interventions as a consequence of comorbid medical problems, concomitant drug therapies, different drug metabolism, higher probability of side effects.

Nonetheless, as described in the limitations section, the possibility to generalize results or to draw clear conclusions about the effectiveness of these interventions is limited. Further methodologically sound studies paying more attention on eligibility criteria, diagnosis, history comorbidity and medications are required to better understand whether a specific type of non-pharmacological intervention should be recommended for depressed elderly in LTCFs.

## Data Availability Statement

The original contributions presented in the study are included in the article/Supplementary Material, further inquiries can be directed to the corresponding author/s.

## Author Contributions

PZ, CG, and MP conceived the study. Review methodology was developed by PZ, CG, MP, and DC. DM and EG performed the database searches and article selection. CG supervised the process. DM, EG, and CG drafted the manuscript. DM, DC, CR, EG, and CG revised the manuscript according to the review's suggestion. All authors revised the draft and contributed with important intellectual content.

## Conflict of Interest

The authors declare that the research was conducted in the absence of any commercial or financial relationships that could be construed as a potential conflict of interest.

## Footnotes

^1^*Modified Jadad Scale*. Available online at: http://www.ohri.ca/programs/clinical_epidemiology/oxford.asp.

## Abbreviations

AAI: animal assisted intervention
AERS: Apparent Emotional Rating Scale
BDI-II: Beck Depression Inventory II
BHS: Beck Hopelessness Scale
BOMCT: Brief Orientation-Memory-Concentration Test
BSI: Beck Suicide Ideation Scale
BTOT: Benton Temporal Orientation Test
CES-D: Centre for Epidemiologic Studies Depression Scale
CSDD: Cornell Scale for Depression in Dementia
DASS 21: 21-item Depression Anxiety Stress Scale
DAT: Dog-assisted Therapy
DI: Depression Inventory
DS: Geriatric Depression Screening Scale
ESC: Emotional Symptom Checklist
GDS-30: 30-item Geriatric Depression Scale
GDS-15: 15-item Geriatric Depression Scale
GDS8: 8-item Geriatric Depression Scale
HAM-D: Hamilton Rating Scale for Depression
HS: Hopelessness scale
ICT: information and communications technology
LSI-A: Life Satisfaction Index A
MINI: Mini international neuropsychiatric interview
MMSE: Mini Mental State Examination
MPCR: Raven's Colored Progressive Matrices
MSQ: Mental Status Questionnaire
NE: Non-experimental
NOS: Newcastle-Ottawa Scale
PANAS: Positive and Negative Affect Schedule
PHQ-9: Patient Health Questionnaire 9
PROMIS: Patient Reported Outcomes Measurement Information System depression scale
QED: Quasi Experimental Design
RCT: Randomized Controlled Trial
SCID: Structured Clinical Interview for DSM-IV
SMMTE: Standardized Mini-Mental Test Examination
SPMSQ: Short Portable Mental Status Questionnaire
TDQ: Taiwanese Depression Questionnaire.
