# Supplementary material for: Non-pharmacological Approaches to Depressed Elderly With No or Mild Cognitive Impairment in Long-Term Care Facilities. A Systematic Review of the Literature
Source: Front Public Health. 2021 Jul 16;9:685860. doi: 10.3389/fpubh.2021.685860 (PMC8322575; doi:10.3389/fpubh.2021.685860)
Supplement: Supplementary file 1 [file Data_Sheet_1.docx]

Supplementary material Table 1. Horticolture and gardening studies: Effect size estimation.

| Horticolture and gardening studies, SMD | | |  |  |  |  |  |
| --- | --- | --- | --- | --- | --- | --- | --- |
| **Study** | **Experimental** | | | **Control** | | | **Std. Mean Difference** |
|  | **mean** | **sd** | **n** | **mean** | **sd** | **n** | **IV, Fixed, 95% CI** |
| Chu, 2019 GDS-15 | 2.71 | 0.32 | 75 | 8.08 | 0.38 | 75 | -15.21 [-16.98, -13.44] |
| Park, 2016 GDS-15 | 4.7 | 2.7 | 24 | 5.1 | 2.9 | 26 | -0.14 [-0.70, 0.42] |
| Lai, 2018 GDS-15 | . | . | . | . | . | . | Not estimable |

Acronyms: GDS Geriatric Depression Scale; SMD Standardized Mean Difference

Supplementary material Figure 1. Horticolture and gardening studies, SMD


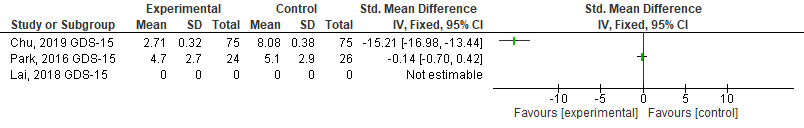


Acronyms: GDS Geriatric Depression Scale; SMD Standardized Mean Difference

Supplementary material Table 2. Pet therapy studies: Effect size estimation.

| Pet therapy studies, SMD |  |  |  |  |  |  |  |
| --- | --- | --- | --- | --- | --- | --- | --- |
| **Study** | **Experimental** | | | **Control** | | | **Std. Mean Difference** |
|  | **mean** | **Sd** | **n** | **mean** | **sd** | **n** | **IV, Fixed, 95% CI** |
| Ambrosi, 2018 GDS-15 | -3.35 | 2.1 | 17 | -0.25 | 2.26 | 12 | -1.39 [-2.22, -0.56] |
| Sollami, 2017 GDS-15 (pre vs post) GDS-15 | -1.57 | 2.7 | 14 | 0.57 | 0.94 | 14 | -1.03 [-1.82, -0.23] |
| Phelps, 2008 GDS-15 | 12 | . | 5 | 11.8 | . | 5 | Not estimable |
| Stasi, 2004 GDS-15 | 7.9 | 4.6 | 14 | 0 | . | 14 | Not estimable |

Acronyms: GDS Geriatric Depression Scale; SMD Standardized Mean Difference

Supplementary material Figure 2. Pet therapy studies, SMD


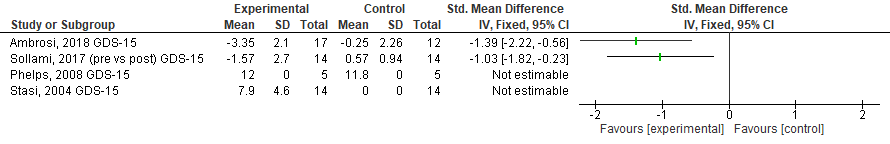


Acronyms: GDS Geriatric Depression Scale; SMD Standardized Mean Difference

Supplementary material Table 3. Physical exercise studies: Effect size estimation.

| Physical exercise studies, SMD |  |  |  |  |  |  |  |
| --- | --- | --- | --- | --- | --- | --- | --- |
| **Study** | **Experimental** | | | **Control** | | | **Std. Mean Difference** |
|  | **mean** | **sd** | **n** | **mean** | **sd** | **n** | **IV, Fixed, 95% CI** |
| Coelho, 2020 GDS-27 | 7.45 | 4.43 | 20 | 12.21 | 5.9 | 19 | -0.90 [-1.56, -0.24] |
| De Carvalho Bastone, 2004 GDS | 9.89 | 4.57 | 19 | 14.06 | 5.4 | 18 | -0.82 [-1.49, -0.14] |
| Brown,2009 (IG1 vs IG2) GDS | 5.3 | 3.8 | 82 | 8.5 | 4.8 | 34 | -0.77 [-1.18, -0.36] |
| Chen, 2015 (3 mo) TDQ | 4.92 | 5.73 | 64 | 10.96 | 9.83 | 63 | -0.75 [-1.11, -0.39] |
| Chen, 2015 (6 mo) TDQ | 5.44 | 7.8 | 64 | 11.53 | 9.85 | 63 | -0.68 [-1.04, -0.32] |
| Chen, 2010 (6 mo) TDQ | 3.03 | 6.71 | 31 | 7.29 | 6.5 | 24 | -0.63 [-1.18, -0.09] |
| Brown,2009 (IG1 vs IG2) PANAS-P | -33.4 | 7.1 | 82 | -29.6 | 5.7 | 34 | -0.56 [-0.97, -0.15] |
| Roswiyani, 2019 (IG3) BDI - II | 7.0 | 6.93 | 67 | 10.92 | 11.1 | 65 | -0.42 [-0.77, -0.08] |
| Roswiyani, 2019 (IG2) BDI - II | 7.84 | 6.9 | 67 | 10.92 | 11.1 | 65 | -0.33 [-0.68, 0.01] |
| Roswiyani, 2019 (IG1) BDI - II | 8.17 | 7.83 | 63 | 10.92 | 11.1 | 65 | -0.28 [-0.63, 0.06] |
| Chao, 2014 (PRE VS POST) GDS-15 | 2.3 | 2.1 | 7 | 2.9 | 2.1 | 7 | -0.27 [-1.32, 0.79] |
| Brown,2009 (IG1 vs IG2) PANAS-N | 13.9 | 5.7 | 82 | 15.3 | 4.2 | 34 | -0.26 [-0.66, 0.14] |
| Brown, 2009 (IG1) PANAS-P | -33.4 | 7.1 | 82 | -31.8 | 7.9 | 38 | -0.22 [-0.60, 0.17] |
| Brown, 2009 (IG1) GDS | 5.3 | 3.8 | 82 | 6.2 | 5.3 | 38 | -0.21 [-0.59, 0.18] |
| Brown, 2009 (IG1) PANAS-N | 13.9 | 5.7 | 82 | 15.1 | 6.0 | 38 | -0.21 [-0.59, 0.18] |
| Vankova, 2014 GDS-15 | 5.0 | 3.29 | 79 | 5.27 | 3.27 | 83 | -0.08 [-0.39, 0.23] |
| Chen, 2010 (3 mo) TDQ | 4.74 | 8.16 | 31 | 5.0 | 4.39 | 24 | -0.04 [-0.57, 0.50] |
| Brown, 2009 (IG2) PANAS-N | 15.3 | 4.2 | 34 | 15.1 | 6.0 | 38 | 0.04 [-0.42, 0.50] |
| Roswiyani, 2019 (IG1 VS IG2) BDI - II | 8.17 | 7.83 | 63 | 7.84 | 6.9 | 67 | 0.04 [-0.30, 0.39] |
| Winningham, 2003 GDS | 5.6 | 5.06 | 25 | 5.18 | 5.06 | 17 | 0.08 [-0.53, 0.70] |
| Roswiyani, 2019 (IG1 VS IG3) BDI - II | 7.84 | 6.9 | 67 | 7.0 | 6.93 | 67 | 0.12 [-0.22, 0.46] |
| Roswiyani, 2019 (IG2 VS IG3) BDI - II | 7.84 | 6.9 | 67 | 7.0 | 6.93 | 67 | 0.12 [-0.22, 0.46] |
| Brown, 2009 (IG2) PANAS-P | -29.6 | 5.7 | 34 | -31.8 | 7.9 | 38 | 0.31 [-0.15, 0.78] |
| Brown, 2009 (IG2) GDS | 8.5 | 4.8 | 34 | 6.2 | 5.3 | 38 | 0.45 [-0.02, 0.92] |

Acronyms: BDI Beck Depression Inventory; GDS Geriatric Depression Scale; IG Intervention Group; PANAS Positive and Negative Affect Schedule (P Positive, N negative); SMD Standardized Mean Difference; TDQ Taiwanese Depression Questionnaire

Supplementary material Figure 3. Physical exercise studies, SMD


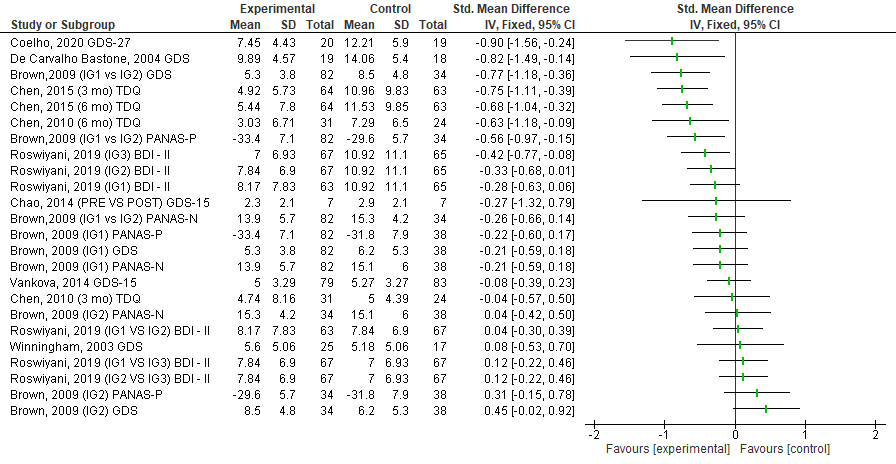


Acronyms: BDI Beck Depression Inventory; GDS Geriatric Depression Scale; IG Intervention Group; PANAS Positive and Negative Affect Schedule (P Positive, N negative); SMD Standardized Mean Difference; TDQ Taiwanese Depression Questionnaire

Supplementary material Table 4. Psychoeducative / Rehabilitation Interventions studies: Effect size estimation (using Review Manager 5.4).

| Psychoeducative / Rehabilitation  Interventions studies, SMD |  |  |  |  |  |  |  |
| --- | --- | --- | --- | --- | --- | --- | --- |
| **Study** | **Experimental** | | | **Control** | | | **Std. Mean Difference** |
|  | **mean** | **sd** | **n** | **mean** | **sd** | **n** | **IV, Fixed, 95% CI** |
| Sok, 2019 GDS | 4.92 | 2.14 | 36 | 10.43 | 1.85 | 37 | -2.73 [-3.37, -2.08] |
| Hsu, 2019 GDS | 2.69 | 2.23 | 40 | 7.58 | 3.95 | 39 | -1.51 [-2.02, -1.01] |
| Dolu, 2018 (8 weeks) GDS | 3.61 | 2.5 | 23 | 8.3 | 4.15 | 24 | -1.34 [-1.98, -0.70] |
| Dolu, 2018 (12 weeks) GDS | 3.68 | 2.53 | 22 | 8 | 4.3 | 22 | -1.20 [-1.85, -0.56] |
| Yuen, 2008 (3 mo) GDS | 4.92 | 4.25 | 15 | 9 | 7.79 | 13 | -0.64 [-1.41, 0.12] |
| Yuen, 2008 (post-intervention) GDS | 5.27 | 3.58 | 15 | 8.38 | 6.68 | 13 | -0.58 [-1.34, 0.18] |
| Tsai, 2008 (2 mo) GDS | 10 | 3.6 | 31 | 12 | 4.9 | 32 | -0.46 [-0.96, 0.04] |
| Rosen, 1997 HAM-D | 9.2 | 2.9 | 11 | 10.9 | 4.3 | 11 | -0.45 [-1.29, 0.40] |
| Tsai,2011 (12 mo) GDS-30 | 13 | 4.5 | 28 | 15.04 | 4.61 | 27 | -0.44 [-0.98, 0.09] |
| Tsai,2011 (6 mo) GDS-30 | 12.85 | 5.35 | 33 | 14.41 | 4.93 | 29 | -0.30 [-0.80, 0.20] |
| Dozeman, 2011 (int completers vs cg) CES-D | 12.2 | 4.8 | 14 | 13.8 | 7.6 | 14 | -0.24 [-0.99, 0.50] |
| Rosen, 1997 GDS | 15.4 | 6.6 | 11 | 16.6 | 5.9 | 11 | -0.18 [-1.02, 0.65] |
| Llewellyn – Jones, 1999 GDS | 11.81 | 4.7 | 86 | 12.57 | 4.1 | 83 | -0.17 [-0.47, 0.13] |
| Dozeman, 2011 (int vs cg) CES-D | 13.5 | 5.7 | 51 | 13.8 | 7.6 | 14 | -0.05 [-0.64, 0.54] |
| Leontjevas, 2013 DU CSDD | Not estimable; authors reported: effect size (0.3); 95%IC (-0.3 to 0.9); p-value 0.379 | | | | | | |
| Leontjevas, 2013 DU GDS | Not estimable; authors reported: effect size (-0.3); 95%IC (-0.7 to 0.1); p-value 0.172 | | | | | | |
| Leontjevas, 2013 SU CSDD | Not estimable; authors reported: effect size (-0.8); 95%IC (-1.4 to 0.1); p-value 0.018 | | | | | | |
| Leontjevas, 2013 SU GDS | Not estimable; authors reported: effect size (-0.1); 95%IC (-0.4 to 0.2); p-value 0.404 | | | | | | |
| Mc Curren, 1999 (12 weeks) GDS-30 | -5.027 | . | 44 | -0.176 | . | 41 | Not estimable |
| Mc Curren, 1999 (24 weeks) GDS-30 | -6.875 | . | 44 | 0.222 | . | 41 | Not estimable |
| Reinhrdt, 2014 (adj means) HAM-D | 6.48 | . | 21 | 3.62 |  | 16 | Not estimable |
| Reinhrdt, 2014 (adj means) PROMIS | 6.8 | . | 21 | 1.53 | . | 16 | Not estimable |
| Supiano, 1989 AB | 9 | . | 62 | 7.8 | . | 54 | Not estimable |
| Supiano, 1989 GDS | 6.2 | . | 62 | 6.1 | . | 54 | Not estimable |
| Tsai,2011 (3 mo) GDS-30 | 11.57 | 5.27 | 35 | 10.56 | 3.89 | 41 | 0.22 [-0.23, 0.67] |
| Cernin, 2009 GDS-30 | 8.9 | 5 | 8 | 7.31 | 6.06 | 7 | 0.27 [-0.75, 1.29] |
| Tsai, 2008 (4 weeks) GDS | 12.8 | 4.9 | 31 | 11.3 | 4.9 | 32 | 0.30 [-0.19, 0.80] |
| Cesetti, 2017 GDS-15 | 6.1 | 3.79 | 20 | 4.3 | 3.16 | 10 | 0.49 [-0.28, 1.26] |

Acronyms: CES-D Centre for Epidemiologic Studies Depression Scale; CSDD Cornell Scale for Depression in Dementia; GDS Geriatric Depression Scale; HAM-D: Hamilton Rating Scale for Depression; PROMIS Patient Reported Outcomes Measurement Information System depression scale; SMD Standardized Mean Difference

Supplementary material Figure 4. Psychoeducative / Rehabilitation Interventions studies, SMD


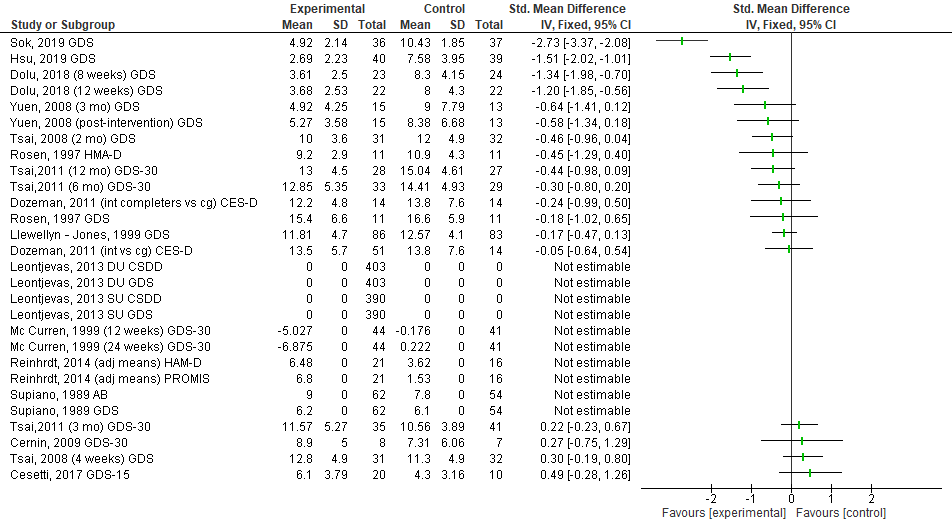


Acronyms: CES-D Centre for Epidemiologic Studies Depression Scale; CSDD Cornell Scale for Depression in Dementia; GDS Geriatric Depression Scale; HAM-D: Hamilton Rating Scale for Depression; PROMIS Patient Reported Outcomes Measurement Information System depression scale; SMD Standardized Mean Difference

Supplementary material Table 5. Psychotherapy studies: Effect size estimation (using Review Manager 5.4).

| Psychotherapy studies, SMD |  |  |  |  |  |  |  |
| --- | --- | --- | --- | --- | --- | --- | --- |
| **Study** | **Experimental** | | | **Control** | | | **Std. Mean Difference** |
|  | **mean** | **sd** | **n** | **mean** | **Sd** | **n** | **IV, Fixed, 95% CI** |
| Hyer, 2009 (post-time 1) GDS-15 | 5 | 3.5 | 13 | 10.5 | 3.6 | 12 | -1.50 [-2.40, -0.59] |
| Konnert, 2009 (6 mo) GDS-30 | 9.44 | 2.71 | 20 | 12.31 | 2.25 | 23 | -1.14 [-1.79, -0.49] |
| Konnert, 2009 (post test) GDS-30 | 10.11 | 2.45 | 20 | 12.5 | 2.58 | 23 | -0.93 [-1.56, -0.30] |
| Konnert, 2009 (3 mo) CESD | 31.59 | 10.3 | 20 | 38.28 | 8.49 | 23 | -0.70 [-1.32, -0.08] |
| Konnert, 2009 (post test) CESD | 31.94 | 7.17 | 20 | 38.22 | 10.09 | 23 | -0.70 [-1.31, -0.08] |
| Konnert, 2009 (3 mo) GDS-30 | 10.83 | 2.81 | 20 | 12.31 | 2.3 | 23 | -0.57 [-1.18, 0.04] |
| Konnert, 2009 (6 mo) CESD | 32.18 | 12.9 | 20 | 36.78 | 9.48 | 23 | -0.40 [-1.01, 0.20] |
| Blair, 2016 BDI-II | . | . | . | . | . | . | Not estimable |
| Blair, 2016 BHS | . | . | . | . | . | . | Not estimable |
| Blair, 2016 DASS 21 | . | . | . | . | . | . | Not estimable |

Acronyms: BDI Beck Depression Inventory; BHS Beck Hopelessness Scale; CES-D Centre for Epidemiologic Studies Depression Scale; DASS Depression Anxiety Stress Scale; GDS Geriatric Depression ScaleSMD Standardized Mean Difference

Supplementary material Figure 5. Psychotherapy studies, SMD


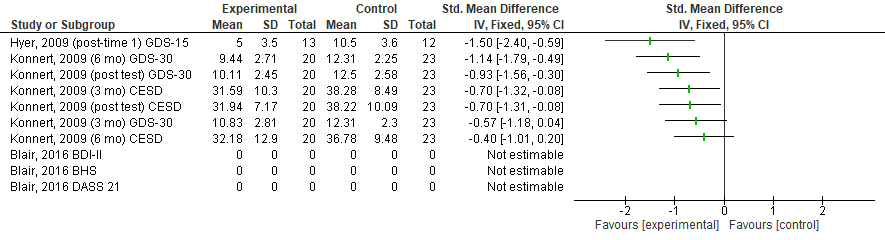


Acronyms: BDI Beck Depression Inventory; BHS Beck Hopelessness Scale; CES-D Centre for Epidemiologic Studies Depression Scale; DASS Depression Anxiety Stress Scale; GDS Geriatric Depression ScaleSMD Standardized Mean Difference

Supplementary material Table 6. Reminiscence & Story Sharing studies: Effect size estimation (using Review Manager 5.4).

| Reminiscence & Story Sharing studies, SMD |  |  |  |  |  |  |  |
| --- | --- | --- | --- | --- | --- | --- | --- |
| **Study** | **Experimental** | | | **Control** | | | **Std. Mean Difference** |
|  | **mean** | **Sd** | **n** | **mean** | **sd** | **n** | **IV, Fixed, 95% CI** |
| Lan, 2018 GDS-15 | 3 | 3 | 31 | 5516 | 3171 | 31 | -2.43 [-3.09, -1.76] |
| Hamzehzadeh, 2018 (8 session) GDS-30 | 7.7 | 2.9 | 15 | 13.2 | 0.75 | 12 | -2.40 [-3.42, -1.37] |
| Hamzehzadeh, 2018 (4 session) GDS-30 | 7.3 | 3.1 | 15 | 12.8 | 1.1 | 12 | -2.19 [-3.18, -1.20] |
| Chueh, 2013 (first 1 wk) | 10.09 | 4.06 | 11 | 19.8 | 4.73 | 10 | -2.12 [-3.24, -1.01] |
| Chiang, 2010 (F-UP) CES-D | 15.49 | 1.99 | 65 | 19.43 | 2.22 | 65 | -1.86 [-2.27, -1.44] |
| Hsu, 2009 GDS-15 | 7.9 | 1.7 | 21 | 10.7 | 2.3 | 24 | -1.35 [-2.00, -0.69] |
| Chueh, 2013 (second 6 mo) GDS-30 | 13.86 | 6.82 | 7 | 22.33 | 4.46 | 6 | -1.34 [-2.60, -0.09] |
| Karimi, 2010 (Integrative vs CG) GDS-15 | 4.7 | 2.62 | 10 | 8 | 2.61 | 10 | -1.21 [-2.18, -0.24] |
| Chueh, 2013 (second 3 mo) GDS-30 | 11.91 | 5.72 | 11 | 18.29 | 3.95 | 7 | -1.18 [-2.23, -0.14] |
| Hamzehzadeh, 2018 (1 mo) GDS-30 | 8.7 | 4.1 | 15 | 12.5 | 1.3 | 12 | -1.16 [-1.99, -0.33] |
| Chiang, 2010 (POST-TEST) CES-D | 16.18 | 2.07 | 65 | 18.74 | 2.7 | 65 | -1.06 [-1.43, -0.69] |
| Chao, 2006 GDS-15 | 2.91 | 2.77 | 12 | 4.63 | 2 | 12 | -0.69 [-1.52, 0.14] |
| Karimi, 2010 (Instrumental vs CG) GDS-15 | 7 | 3.31 | 9 | 8 | 2.61 | 10 | -0.32 [-1.23, 0.59] |
| Karimi, 2010 (Integrative vs Instrumental) GDS-15 | 7 | 3.31 | 9 | 8 | 2.61 | 10 | -0.32 [-1.23, 0.59] |
| Jones, 2003 GDS-30 | 12.13 | 4.61 | 15 | 12.46 | 3.96 | 15 | -0.07 [-0.79, 0.64] |
| Sullivan, 2019 DI | 15.85 | 6.16 | 48 | 16.17 | 6.66 | 52 | -0.05 [-0.44, 0.34] |
| Chuang, 2018 GDS-15 | . | . | 35 | . | . | 35 | Not estimable |
| Haight, 2000 | . | . | 29 | . | . | 23 | Not estimable |
| Meléndez-Moral, 2013 | . | . | 17 | . | . | 17 | Not estimable |
| Zauszniewski, 2004 ESC | . | . | . | . | . | . | Not estimable |
| Westerhof, 2017 (8 mo) GDS-8 | 1.6 | 2.2 | 42 | 1.3 | 1.8 | 39 | 0.15 [-0.29, 0.58] |
| Westerhof, 2017 (2 mo) GDS-8 | 1.4 | 2 | 42 | 1.1 | 1.5 | 39 | 0.17 [-0.27, 0.60] |

Acronyms: CES-D Centre for Epidemiologic Studies Depression Scale; DI Depression Inventory; ESC: Emotional Symptoms Checklist; GDS Geriatric Depression Scale; SMD Standardized Mean Difference

Supplementary material Figure 6. Reminiscence & Story Sharing studies, SMD


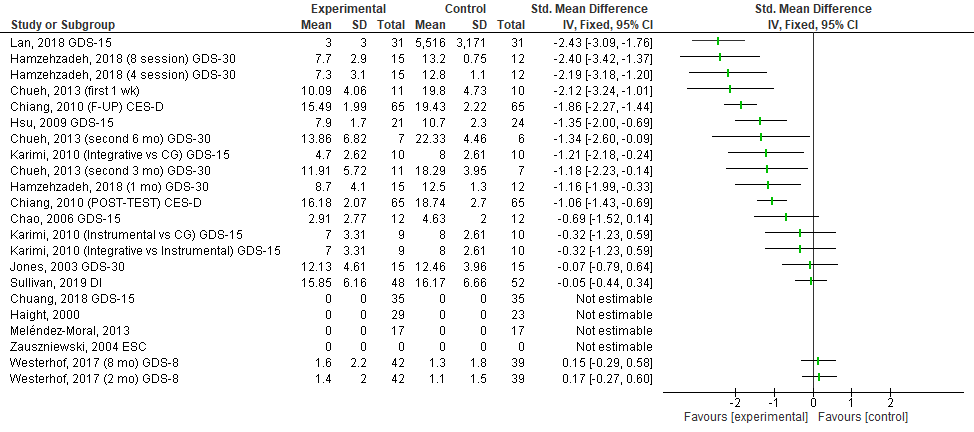


Acronyms: CES-D Centre for Epidemiologic Studies Depression Scale; DI Depression Inventory; ESC: Emotional Symptoms Checklist; GDS Geriatric Depression Scale; SMD Standardized Mean Difference

Supplementary material Table 7. Miscellaneous articles: Effect size estimation (using Review Manager 5.4).

| Miscellaneous articles, SMD |  |  |  |  |  |  |  |
| --- | --- | --- | --- | --- | --- | --- | --- |
| **Study** | **Experimental** | | | **Control** | | | **Std. Mean Difference** |
|  | **mean** | **sd** | **n** | **mean** | **sd** | **n** | **IV, Fixed, 95% CI** |
| Han, Ji Hyoung, 2017 GDS-15 | -3.05 | 1.31 | 19 | -0.22 | 1.17 | 18 | -2.23 [-3.06, -1.39] |
| Chiu, 2019 (ICT-Ent vs CG) CES-D | 5.78 | 3.27 | 18 | 11.76 | 4.64 | 17 | -1.46 [-2.22, -0.71] |
| Biasutti, 2019 GDS-15 | 3.6 | 2.72 | 20 | 4.84 | 3.01 | 25 | -0.42 [-1.02, 0.17] |
| Wang, 2011 GDS-15 | 3.77 | 3.17 | 35 | 4.66 | 4.06 | 41 | -0.24 [-0.69, 0.21] |
| Chiu, 2019 (ICT-Comm vs CG) CES-D | 11 | 3.27 | 19 | 11.76 | 4.64 | 17 | -0.19 [-0.84, 0.47] |
| Rajagopal, 2002 (group pre-post) CES-D | 19.29 | . | 14 | 22.93 | . | 14 | Not estimable |
| Rajagopal, 2002 (individual pre-post) CES-D | . | . | 8 | . | . | 8 | Not estimable |
| Bosmans, 2013 CES-D | -3.2 | . | 93 | -0.8 | . | 92 | Not estimable |
| Chang, 2010 GDS-15 | . | . | 10 | . | . | 10 | Not estimable |
| Tai, 2015 (1 mo) GDS-SF | 2.83 | 2.19 | 41 | 2.63 | 2.22 | 19 | 0.09 [-0.45, 0.63] |
| Tai, 2015 (4 mo) GDS-SF | 0.26 | 0.59 | 27 | 0.13 | 0.34 | 16 | 0.25 [-0.37, 0.87] |
| Chiu, 2019 (ICT-Comm vs ICT-Ent) CES-D | 11 | 3.27 | 19 | 5.78 | 3.27 | 18 | 1.56 [0.82, 2.31] |

Acronyms: CES-D Centre for Epidemiologic Studies Depression Scale; CG Control Group; DI Depression Inventory; ESC: Emotional Symptoms Checklist; GDS Geriatric Depression Scale; ICT Information and Communications Technology; SMD Standardized Mean Difference

Supplementary material Figure 7. Miscellaneous articles, SMD


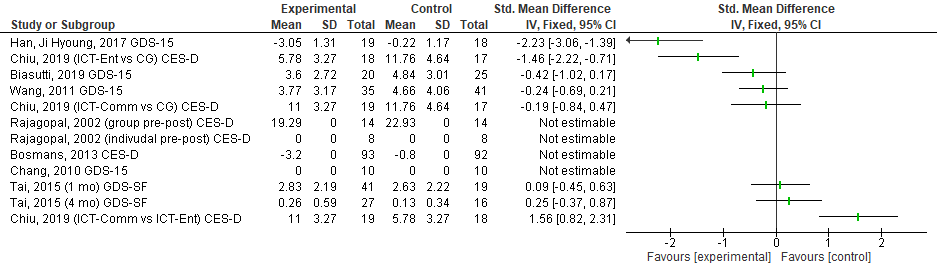


Acronyms: CES-D Centre for Epidemiologic Studies Depression Scale; CG Control Group; DI Depression Inventory; ESC: Emotional Symptoms Checklist; GDS Geriatric Depression Scale; ICT Information and Communications Technology; SMD Standardized Mean Difference
